# Preparation, characterization, pharmacokinetics and anticancer effects of PEGylated β-elemene liposomes

**DOI:** 10.20892/j.issn.2095-3941.2019.0156

**Published:** 2020-02-15

**Authors:** Bingtao Zhai, Qibiao Wu, Wengang Wang, Mingming Zhang, Xuemeng Han, Qiujie Li, Peng Chen, Xiaying Chen, Xingxing Huang, Guohua Li, Qin Zhang, Ruonan Zhang, Yu Xiang, Shuiping Liu, Ting Duan, Jianshu Lou, Tian Xie, Xinbing Sui

**Affiliations:** ^1^College of Pharmacy, Chengdu University of Traditional Chinese Medicine, Chengdu 611137, China; ^2^Department of Holistic Integrative Pharmacy Institutes and Comprehensive Cancer Diagnosis and Treatment Center, the Affiliated Hospital of Hangzhou Normal University, College of Medicine, Hangzhou Normal University, Hangzhou 310018, China; ^3^State Key Laboratory of Quality Research in Chinese Medicines, Faculty of Chinese Medicine, Macau University of Science and Technology, Macau 519020, China; ^4^Key Laboratory of Elemene Class Anticancer Chinese Medicine of Zhejiang Province and Engineering Laboratory of Development and Application of Traditional Chinese Medicine from Zhejiang Province, Hangzhou Normal University, Hangzhou 310018, China

**Keywords:** β-elemene, PEGylated liposome, pharmacokinetics, antitumor effect, bladder cancer

## Abstract

**Objective:** This study aimed to develop a new polyethylene glycol (PEG)ylated β-elemene liposome (PEG-Lipo-β-E) and evaluate its characterization, pharmacokinetics, antitumor effects and safety *in vitro* and *in vivo*.

**Methods:** The liposomes were prepared by ethanol injection and high-pressure micro-jet homogenization. Characterization of the liposomes was conducted, and drug content, entrapment efficiency (EE), *in vitro* release and stability were studied by ultra-fast liquid chromatography (UFLC) and a liquid surface method. Blood was drawn from rats to establish the pharmacokinetic parameters. The anticancer effect was evaluated in a KU-19-19 bladder cancer xenograft model. Histological analyses were performed to evaluate safety.

**Results:** The PEG-Lipo-β-E showed good stability and was characterized as 83.31 ± 0.181 nm in size, 0.279 ± 0.004 in polydispersity index (PDI), −21.4 ± 1.06 mV in zeta potential, 6.65 ± 0.02 in pH, 5.024 ± 0.107 mg/mL in β-elemene (β-E) content, and 95.53 ± 1.712% in average EE. The Fourier transform infrared spectroscopy (FTIR) and differential scanning calorimetry (DSC) indicated the formation of PEG-Lipo-β-E. Compared to elemene injection, PEG-Lipo-β-E demonstrated a 1.75-fold decrease in clearance, a 1.62-fold increase in half-life, and a 1.76-fold increase in area under the concentration-time curves (AUCs) from 0 hour to 1.5 hours (*P* < 0.05). PEG-Lipo-β-E also showed an enhanced anticancer effect *in vivo*. Histological analyses showed that there was no evidence of toxicity to the heart, kidney, liver, lung or spleen.

**Conclusions:** The present study demonstrates PEG-Lipo-β-E as a new formulation with ease of preparation, high EE, good stability, improved bioavailability and antitumor effects.

## Introduction

Elemenes are a group of natural compounds extracted from the herb *Curcumae Rhizoma*. Elemene contains 3 major components: α-, β-, and δ-elemene. Among them, β-elemene (β-E) has the highest antitumor activity^1^. The chemical name of β-E is 1-methyl-1-vinyl-2,4-diisopropenyl-cyclohexane, the molecular formula is C_15_H_24_, and the molecular weight (MW) is 204.35 g/mol ^2^. β-E has been shown to inhibit tumor cell growth by targeting the Wnt/β-catenin^3–6^, Notch^7–9^, PI3K/AKT/mTOR^10–12^, MAPK/ERK^13–15^, NF-κB^16^ and STAT3 pathways^17–19^. β-E also increases the sensitivity of tumor cells to chemoradiotherapy, reverses chemotherapy multiple-drug resistance and regulates tumor-associated immune responses^20–23^.

However, the clinical application of elemene has been limited by its poor solubility in water, poor stability, and low bioavailability^24^. Currently, an elemene emulsion injection and an oral emulsion have been developed to treat various cancers in the clinic for > 20 years. Although the emulsion increases the bioavailability of β-E, it is easily cleared by the kidneys and taken up by the reticuloendothelial system (RES) residing in the liver and the spleen because of the small particle size. Some symptoms may also occur upon oral or intravenous (iv) administration, including phlebitis, fever, pain, allergy, bleeding, nausea, vomiting, leukocytopenia, baldness, and liver dysfunction^25^. Therefore, it is necessary to study novel formulations for β-E to extend its plasma circulation lifetime and to enhance its therapeutic activity at doses exhibiting acceptable toxicities.

In the area of cancer therapy, several polymers have successfully been used as carriers for sustained drug delivery. For example, polyethylene glycol (PEG) and its derivatives such as mono methoxy poly (ethylene glycol)-poly (e-caprolactone) (mPEG-PCL), and poly lactic-co-glycolic acid (PLGA). These copolymers are not accumulative *in vivo* because they can be eliminated by the kidneys or their degradation products can enter the tricarboxylic acid cycle^26–29^. Among the different ways investigated in the attempt to improve the blood circulation time of anticancer drugs, anchoring the PEG on the surface of drug delivery systems is the most widely used method, especially on the surface of liposomes.

Liposomes are spherical vesicles created by a lipid bilayer of phospholipids. Due to their good biocompatibility, weak immunogenic response, and low intrinsic toxicity, liposomes have become one of the best choices for pharmaceutical carriers^30^. Recently, to improve the antitumor effects and to overcome the shortcomings of conventional vesicles being easily adsorbed by plasma proteins and rapidly cleared by organs such as the liver and spleen, researchers have obtained long-circulating liposomes by grafting the particle surface with PEG, which shields the vesicles from interaction with macrophages and creates a long circulating “stealth” effect. For instance, DOXIL/Caelyx is a PEGylated liposomal doxorubicin, which is composed of hydrogenated soybean phospholipids, cholesterol, mPEG2000-DSPE and has been approved in both the USA and Europe for the treatment of Kaposi’s sarcoma and recurrent ovarian cancer^31^. The long circulation time and improved stability allow the vesicles to extravasate to the tumor site and be retained for days^32–34^. In this study, we aimed to develop a PEGylated β-E liposome to prolong the half-life of the liposomes in plasma and enhance their anticancer effects. PEG-Lipo-β-E was prepared, and the physical properties such as particle size, zeta potential, polydispersity index (PDI) and others were evaluated. In addition, its pharmacokinetics, anticancer effects and safety were investigated to estimate the potential efficacy for clinical use.

## Materials and methods

### Materials

β-E standard (99.4% pure; National Institute for the Control of Pharmaceutical and Biological Products, Beijing, China), β-E crude drug (99.1% pure; Jusheng Technology Co., Hubei, China), elemene injection (0.1 g/20 mL, Dalian Holley King Kong Pharmaceutical Co., Dalian, China), acetonitrile (Merck Co., Ltd, Darmstadt, Germany), soybean lecithin (Tywei, Shanghai, China), cholesterol (99.5% pure, Dingguo Changsheng Biotechnology Co., Beijing, China), Lipoid DSPE-PEG 2000 (Lipoid GmbH, Ludwigshafen, Germany), L-histidine (Sinopharm Chemical Reagent Co., Shanghai, China), Kolliphor^®^ hydroxystearate (HS) 15 (BASF, Ludwigshafen, Germany), 95% ethanol (Sinopharm Chemical Reagent Co., Shanghai, China), cell counting kit 8 (Dalian Meilun Biotechnology Co., Ltd., Dalian, China), RPMI 1640 (Gibco, Waltham, MA, USA), and fetal bovine serum (FBS) (Gibco, Waltham, MA, USA) were obtained. All other reagents used in this study were of analytical grade and were used without further purification. The water used in this study was filtered using a Millipore Milli-Q™ water purification system (Millipore Corp., Milford, MA, USA).

### Rats and mice

Male Sprague Dawley rats and female BALB/c mice (6 to 8 weeks old) were purchased from the Institute of Experimental Animals of the Zhejiang Academy of Medical Sciences and housed in a standard polypropylene cage containing sterile bedding under controlled temperature (23 ± 2) °C and humidity (50% ± 5)%. All experimental procedures were approved by the Medical Ethics Committee of the Hangzhou Normal University (Approval No. 2017052).

### Preparation of PEG-Lipo-β-E

The oil phase containing 0.5 g of β-E, 2.5 g of soybean lecithin, 0.1 g of cholesterol, and 0.2 g of DSPE-PEG 2000 was dissolved in 2 mL of 95% ethanol in an 80 °C water bath. The aqueous phase was prepared by adding 10 mM L-histidine to 100 mL of water, followed by adjusting the pH to 6.5. The oil phase and aqueous phase were mixed slowly at 60 °C, and the liposomes were formed using an Ultra-Turrax T18 high-speed blender (IKA, Staufen, Germany) at 13,700 *g* for 60 minutes. The liposomes were passed through an LM20 microfluidizer (Microfluidics Co., Westwood, MA, USA) at a pressure of 15,000 psi for 3 cycles, and then the solution was filtered through a 0.22 µm filter.

### Characterization of PEG-Lipo-β-E

The morphology of PEG-Lipo-β-E was determined using a transmission electron microscope (TEM) (HT-7700, Hitachi, Japan). The mean particle size, zeta potential and PDI of liposome droplets were determined by a Zetasizer Nano ZS (Malvern Instruments, Malvern, UK). The pH values of the samples were measured using a pH meter at (20 ± 1) °C (PB-10, Sartorius, Goettingen, Germany).

### Fourier transform infrared spectroscopy (FTIR)

The FTIR spectrum of the pure drug, physical mixture (β-E/soybean lecithin/cholesterol/DSPE-PEG 2000) and the PEG-Lipo-β-E was obtained using an FTIR spectrophotometer (Alpha, Bruker, Germany)^35^. The PEG-Lipo-β-E was freeze-dried before measurement (Alpha 1-2 LD plus, Martin Christ, Germany). The samples were crushed to a fine powder, mulled with anhydrous potassium bromide, pressed to form a thin pellet, and the spectra were obtained in the wavenumber region between 4,000 and 400 cm^−1^.

### Differential scanning calorimetry (DSC)

Thermal analysis of PEG-Lipo-β-E was performed using a differential scanning calorimeter (TA Q200, USA). The samples were weighed, filled in aluminum pans, sealed, and analyzed at a heating rate of 5 °C/minute from 0 to 80 °C. To evaluate any changes during formulation, DSC measurement was also performed for soybean lecithin, cholesterol, PEG-Lipo (blank liposomes) and physical mixture of the formulation components^36^.

### Ultra-fast liquid chromatography (UFLC) analysis of β-E in PEGylated liposomes and rat plasma

According to the literature^37^, the drug content was detected by UFLC (Shimadzu Co., Ltd., Kyoto, Japan). The chromatographic conditions were set as follows: C_18_ column (2.2 µm, 75 mm × 3.0 mm), acetonitrile/water (80:20) as the mobile phase, 40 °C as the column temperature, 210 nm as the detection wavelength, 1.0 mL/minute as the flow rate, and 5 µL as the injection volume.

### Content and entrapment efficiency of PEG-Lipo-β-E

To determine the drug content of PEG-Lipo-β-E, 0.2 mL of the PEGylated liposomes was taken and diluted to 100 mL with an 80% acetonitrile aqueous solution. One milliliter of this sample was determined by UFLC, and this procedure was repeated 3 times.

The EE of β-E in the PEGylated liposomes was determined as previously reported by the liquid surface method^37^. Free β-E was insoluble in water and floated on the surface of the aqueous phase; thus, the internal solution of the liposomes was taken to determine the EE. The sample (2 mL) was moved into a tube and allowed to stand for 2 hours at room temperature. Then, 0.1 mL of the internal solution was removed and diluted to 50 mL with an 80% acetonitrile aqueous solution to be measured for β-E content as “*a*”. The remaining solution (1.9 mL) was also diluted to 50 mL with the 80% acetonitrile aqueous solution. Then, 1 mL of the second diluted sample was removed and diluted again to 25 mL to measure the β-E content as “*b*”. After repeating once as above, the average EE was calculated using the following equations:

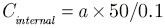

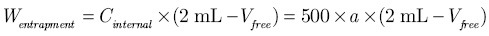

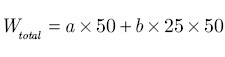

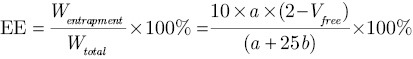









Here, *V_free_* is the volume of β-E at the liquid surface. Because the volume of β-E was found to be insignificant on the surface of PEG-Lipo-β-E (at least 0.05 mL), the EE should be more than 19.5 × *a*/(*a* + 25 *b*) × 100%.

### *In vitro* release of β-E from the PEGylated liposomes

The *in vitro* release profile of β-E from the liposomes was assessed by determining the residual amount of β-E in the liposomes^38^. Briefly, 200 µL of PEG-Lipo-β-E or elemene injection (5 mg/mL) was placed in 5 dialysis bags (MW cutoff 12,000–14,000 Da), ligated, immersed in the 0.5% HS 15 release media (2,500 mL) and stirred by magnetic force at 25 °C. Samples were taken out at predetermined time intervals of 0, 4, 8, 12, 24, and 36 hours. The aqueous dispersion in the dialysis bag was transferred to a 10 mL volumetric flask, and the remains in the bag were washed into the same volumetric flask with a small amount of release media. Then, 5 mL of ethanol was added to the flask to extract the drug dissolved in the release media. The supernatants were filtered through 0.22 µm microporous membranes and subjected to UFLC analysis for the determination of β-E. The *in vitro* release of β-E from the PEGylated liposomes and elemene injection was repeated 3 times.

### Stability studies

Three batches of PEG-Lipo-β-E suspension were prepared according to the mentioned procedure, stored in a 4 °C refrigerator, and removed after 7 days, 15 days, and 30 days. The appearance of the liposomes was observed, and the EE, particle size, and content of β-E were determined.

### Pharmacokinetics of PEG-Lipo-β-E

For the pharmacokinetics study, 10 male Sprague Dawley rats weighing 180–200 g were divided randomly into an elemene injection group (5 rats) and a PEG-Lipo-β-E group (5 rats) for iv injection of β-E (40 mg/kg)^39^. Blood (0.5 mL) was obtained from the ocular vein of the rats at 5, 10, 20, 30, 40, 60, and 90 minutes after iv administration. Then, plasma was obtained after centrifugation at 4,000 × *g* for 10 minutes at 4 °C. A mixture of 0.2 mL of plasma and 0.8 mL of acetonitrile was oscillated for 2 minutes and then centrifuged at 12,000 × *g* for 10 minutes at 4 °C. The supernatant was taken and filtered through a 0.22 µm filter. The filtrate was used for the determination of β-E in plasma.

### *In vitro* cytotoxicity studies in human bladder cancer cells

To determine the cell survival rate after incubation with free β-E, PEG-Lipo-β-E and elemene injection, a CCK-8 assay was carried out using T24 cells and KU-19-19 cells^20^. T24 cells and KU-19-19 cells were incubated in 1640 medium supplemented with 1% (v/v) penicillin/streptomycin and 10% (v/v) FBS. The 2 cells were seeded at a density of 3 **×** 10^4^ cells/well in 96-well plates and incubated at 37 °C and 5% CO_2_ for 24 hours. Culture medium was removed and 25, 50, 75 or 100 µg/mL free β-E, elemene injection or PEG-Lipo-β-E with cell culture medium was added to the wells. RPMI 1640 medium without liposomes was used as a control. After 24 hours or 48 hours incubation, 10 µL of CCK-8 was added to each well. After 4 hours of incubation, absorbance measurements were performed at 450 nm using a microplate reader (Multiskan™ FC, Thermo Scientific™, Waltham, MA, USA).

### *In vitro* cell migration assay

The inhibitory effect of PEG-Lipo-β-E on the migration of bladder cancer cells was measured by wound healing and Transwell migration assays^40^. Bladder cancer KU-19-19 cells were used in this test. For the wound healing assay, cells were seeded at a density of 1.5 × 10^6^ cells per well on 6-well plates and were cultured until they reached 80%–90% confluency. Then, the media was removed, and a sterile 200 µL pipette tip was applied to scratch a vertical wound. The cells were washed with phosphate-buffered saline (PBS) 3 times and incubated with blank PEGylated liposomes, free β-E or PEG-Lipo-β-E (at an equivalent β-E dose of 30 µg/mL for KU-19-19 cells). In contrast, cells that did not receive any treatment after the scratches were used as negative controls. Images were captured at 0 hour and 36 hours after the treatment with an inverted microscope (Carl Zeiss, Jena, Germany).

For the Transwell migration assay, 2 × 10^5^ cells were seeded in the upper chamber of Transwell plates (24-well insert, pore size, 8 µm, Corning, USA). A total of 100 µL of serum-free media with PEG-Lipo, free β-E or PEG-Lipo-β-E (at an equivalent β-E dose of 30 µg/mL) was added into the upper chamber, while 600 µL of complete medium containing 10% FBS was loaded into the lower chamber as a chemoattractant. The cells without any treatment were used as controls. After 36 hours, the upper medium was removed, and a cotton swab was applied to remove the remaining cells. Then, the Transwell chambers were slightly washed in PBS, fixed using 4% paraformaldehyde for 20 minutes and stained with crystal violet for 20 minutes. Finally, the upper chambers were washed with PBS twice, and the stained Transwell chambers were visualized under a microscope (Nikon Eclipse Ci-S, Tokyo, Japan).

### *In vivo* antitumor efficacy

Evaluation of the *in vivo* antitumor efficacy was based on the determination of the tumor volume (TV), calculated as previously described^41^, where TV = 0.5 × (d1 **×** d2^2^), with d1 and d2 being the largest and the smallest perpendicular diameters, respectively. Briefly, KU-19-19 cells [obtained from the American Type Culture Collection (ATCC)] were injected into the abdominal cavity of mice for 6–8 days before they were collected and resuspended in PBS. BALB/c nude female mice were divided into 3 groups (5 mice/group), followed by subcutaneous inoculation with KU-19-19 tumor cells into the right axilla at a volume of 0.2 mL (2.5 **×** 10^6^ cells/mL). After inoculation, groups 1 and 2 were continuously injected with either elemene injection (β-E equivalent dose of 50 mg/kg), PEG-Lipo-β-E (β-E equivalent dose of 50 mg/kg) or PEG-Lipo (blank liposomes) via intraperitoneal injection. Tumor growth was monitored every 4 days. Sixteen days later, all of the animals were sacrificed by cervical dislocation, and the tumors were isolated and weighed. Alterations in the TV were determined by considering the initial volume as 100% and calculating the percent increase or decrease according to this initial volume. The relative tumor volume (RTV) and the percent of tumor growth inhibition (TGI) for each experimental group were calculated using the following equations:

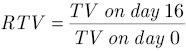

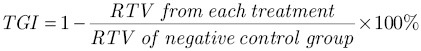





### Histological analysis

After the end of the treatment (day 16), the animals were anesthetized and euthanized. Heart, kidney, liver, lung, and spleen tissues were collected and fixed in paraformaldehyde (4% w/v in PBS) to investigate the toxicity. Then, these tissues were embedded in paraffin blocks, sectioned into 5-µm thick slices, placed onto glass slides, and stained with hematoxylin-eosin. The body weight was measured every 4 days.

### Statistical analysis

The data were statistically analyzed by SPSS Version 17.0 SPSS (SPSS Inc. Chicago, IL, USA). All analyses were carried out in triplicate, and the results are reported as the mean ± standard deviation (SD). Statistical analysis of the pharmacokinetic study was carried out using DAS 2.0 software (Drug and Statistics software, Shaanxi University of Traditional Chinese Medicine, Shaanxi, China). A value of *P* < 0.05 was regarded as a significant difference.

## Results

### Preparation and characterization of PEG-Lipo-β-E

**Figure 1:**
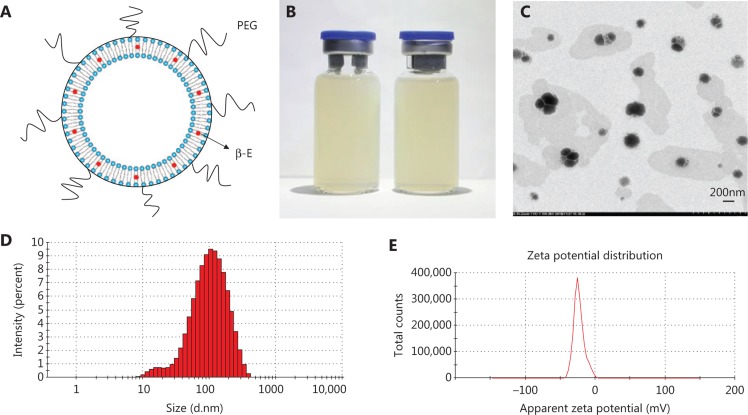
The characterization of PEG-Lipo-β-E. (A) The schematic diagram of PEG-Lipo-β-E. (B) The appearance of PEG-Lipo-β-E. (C) TEM analysis of PEG-Lipo-β-E (5,000 ×). (D) Particle size of PEG-Lipo-β-E. (E) Zeta potential of PEG-Lipo-β-E. PEG, polyethylene glycol; β-E: β-elemene.

In the present study, PEGylated liposomes were prepared by ethanol injection and high-pressure micro-jet homogenization. The schematic diagram of PEG-Lipo-β-E is presented in **Figure 1A**. The ration of soybean lecithin, cholesterol, DSPE-PEG 2000 and β-E was 25:1:2:5. DSPE-PEG 2000 (2%) was added to the liposomes to exert a long-circulating effect. As shown in **Figure 1B**, PEG-Lipo-β-E is a milky white liquid. The TEM imaging shows that the liposomes are round in shape with a uniform size (**Figure 1C**). The mean diameter of the freshly prepared liposomes was 83.31 ± 0.181 nm with a particle diameter size that ranged from 10 to 400 nm (**Figure 1D**), and the PDI value was 0.279 ± 0.004, indicating that its particle size distribution is acceptable and indicating a homogenous population of phospholipid vesicles^42^. The zeta potential of the liposomes was −21.4 ***±*** 1.060 mV, indicating a negatively charged surface on the liposomes (**Figure 1E**). The pH was 6.65 ± 0.020.

### FTIR

**Figure 2:**
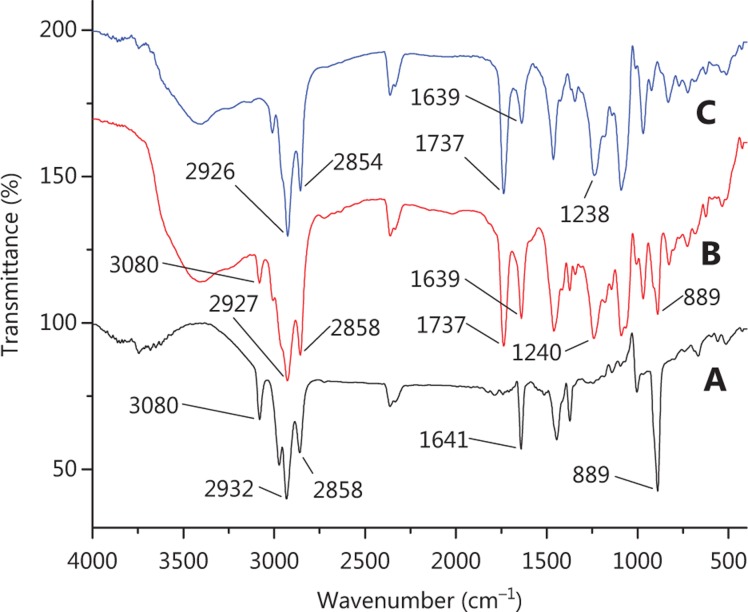
FTIR spectrum of (A) β-E, (B) physical mixture (β-E/soybean lecithin/cholesterol/DSPE-PEG 2000) and (C) PEG-Lipo-β-E.

The FTIR technique can be used to explain the mechanism of encapsulating by exploring the interactions among the liposome components. The FTIR spectra obtained for the pure drug, physical mixture and liposomes are shown in **Figure 2**. It can be observed from the spectra that many peaks related to the β-E (from 3080 to 889 cm^−1^) did not appear in the spectra of PEG-Lipo-β-E, probably due to the drug being encapsulated in the formulations or covered by other ingredients in the liposomes. In the spectra obtained for β-E, the physical mixture and PEG-Lipo-β-E, the bands at 1737 and 1641/1639 cm^−1^ indicated the presence of the C=C, C=O and NH_2_ in the β-E, DSPE and soybean lecithin, but the peak at 1639 cm^−1^ in liposomes was significantly reduced, indicating interaction between liposome components. The asymmetric stretching vibration of the P=O group could be identified by a peak near 1200 cm^−143^. In the spectra obtained for physical mixture and PEG-Lipo-β-E, this peak was at 1240 cm^−1^ and 1230 cm^−1^, respectively. In addition, the peaks at 2858/2854 and 2932/2927/2926 cm^−1^ were present in the spectra of the pure drug, physical mixture and the PEG-Lipo-β-E. These peaks were related to the symmetric and asymmetric CH_2_ stretching vibrations in the aliphatic structure of the PEG, DSPE and β-E. These results preliminarily indicate that β-E is encapsulated in PEGylated liposomes.

### DSC

**Figure 3:**
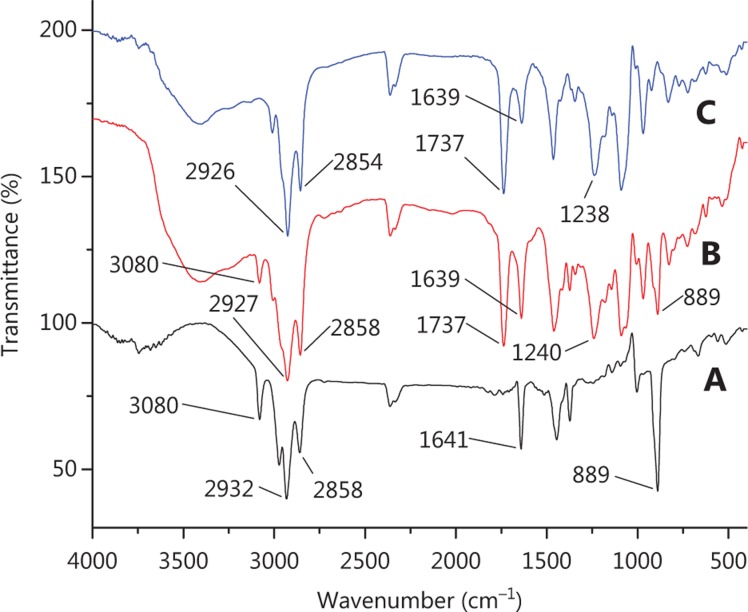
DSC chromatogram of (A) soybean lecithin, (B) cholesterol, (C) physical mixture (β-E/soybean lecithin/cholesterol/DSPE-PEG 2000), (D) PEG-Lipo and (E) PEG-Lipo-β-E.

DSC is a very useful tool to investigate the interaction between liposomes and drug molecules and provides information about the physicochemical state of nanoparticles^44^. A thermogram of soybean lecithin, cholesterol, a physical mixture of β-E/soybean lecithin/cholesterol/DSPE-PEG 2000, PEG-Lipo and PEG-Lipo-β-E are presented in **Figure 3**. As shown in **Figure 3A** and **3B**, soybean lecithin and cholesterol exhibit a single endothermic peak at temperature of 36.83 °C and 42.34 °C, respectively. The DSC curve of PEG-Lipo-β-E exhibits a high-intensity sharp endothermic peak at a temperature of 46.14 °C, which is significantly different from the peak of the soybean lecithin, cholesterol and the physical mixture. This peak is also detected in the thermogram of the PEG-Lipo, although the peak becomes less intensive. These changes can be attributed to the possible interaction between the liposomal components such as soybean lecithin, cholesterol, DSPE-PEG 2000 and the drug.

### Content and entrapment efficiency of PEG-Lipo-β-E

**Figure 4:**
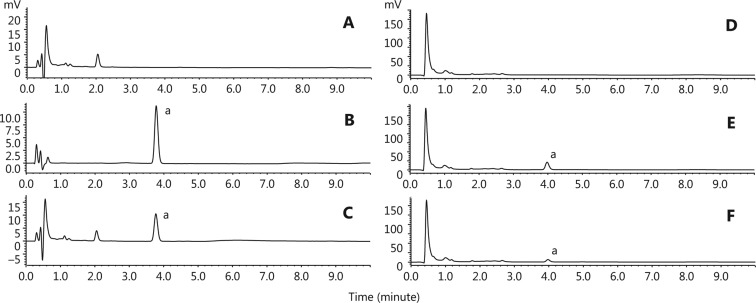
Chromatograms of β-E with ultraviolet (UV). (A) Chromatograms of PEG-Lipo; (B) chromatograms of β-E in mobile phase; (C) chromatograms of β-E in PEGylated liposomes; (D) chromatograms of blank plasma; (E) chromatograms of standard plasma sample; (F) chromatograms of PEG-Lipo-β-E in rat plasma; a, β-E.

In this study, UFLC was used to determine the drug content in the PEGylated liposomes and rat plasma (**Figure 4**). Methodological verification showed that the specificity, linearity, limit of quantitation, precision and accuracy, recovery and matrix effects, stability and durability were all in compliance with the requirements (**Tables 1–4** and **Supplementary Tables S1–S5**). The content of β-E in the PEGylated liposomes was 5.024 ± 0.107 mg/mL, which is equal to the commercial elemene injection. The EE of PEG-Lipo-β-E was obtained by determining the drug concentration of the internal and total solutions using the liquid surface method^37^. The average EE of β-E was (95.53 ± 1.712)%.

### *In vitro* release of β-E from PEGylated liposomes

**Figure 5:**
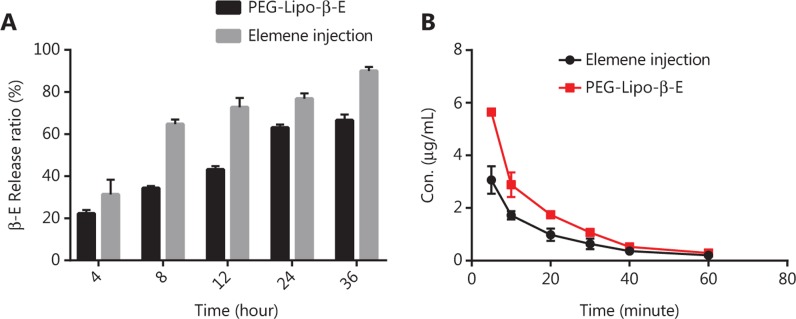
*In vitro* release and the plasma concentration-time curves of elemene injection and PEG-Lipo-β-E. (A) *In vitro* release of β-E from elemene injection and PEG-Lipo-β-E containing 5.024 mg/mL β-E (*n* = 3). (B) The mean plasma concentration of β-E-*vs.*-time curves after intravenous injection of PEG-Lipo-β-E and elemene injection (*n* = 5). Con., concentration.

A PBS pH 7.4 solution containing 0.5% HS 15 was chosen as the dialysis medium for the *in vitro* release of β-E from PEGylated liposomes. The solubility of β-E in the release media could fulfill sink conditions without obviously changing the physical state of the liposomes. The percent of release was calculated by determining the remaining drug in the dialysis bag because of the instability of the drug in the release medium^38^. The *in vitro* release behavior of the conventional injection and PEGylated liposomes was studied, and the results are depicted in **Figure 5A**. The elemene injection showed 90% drug release after 36 hours. The PEGylated liposome showed a drug release of only 66% after 36 hours, and this can be attributed to the slower diffusion of the drug from the liposomes with the addition of DSPE-PEG 2000. There was a sharp increase in the drug release after 24 hours for the conventional injection. The more rapid drug release might be due to leakage of the drug from the lipid bilayer, which acts as a rate-limiting membrane for the release of the encapsulated drug. The drug release profile can reflect the distribution of the drug in the vehicle. Drugs that are not completely encapsulated in the lipid matrix have a burst release. The release profile of β-E from the liposomes was steady during the test time, which suggests that β-E might be homogeneously dispersed in the lipid matrix. The obtained release data were fitted to zero-order, first-order, Higuchi, and Weibull equations^45^, and the results showed that β-E release from the conventional injection and the PEGylated liposomes best fit the Weibull equation (**Table 5**). The overall *R*^2^ values for both the elemene injection and the PEGylated liposomes can be attributed to the Weibull model release pattern. These results indicate that the PEGylated liposomal formulations meet the requirement for a sustained drug delivery system compared to the conventional injection.

### Stability studies

Stability is one of the major obstacles for the formulation of liposomes because of the aggregation and leaching of drugs from the lipid layers. Moreover, liposomes are easy to oxidize because they are made up of lipids; hence, stability is the major problem for liposome storage^46,47^. The physical stability results of PEG-Lipo-β-E for different times are shown in **Table 6**. After a storage period of 30 days, the liposomes showed no significant change in β-E content and physical appearance (no precipitation, no flocculation and delamination, uniform dispersion, good fluidity, and obvious opalescence to light), with a slight change in EE, which decreased by 4% of the original value. The particle size of PEG-Lipo-β-E under storage conditions increased by 2% of the original value. In general, PEG-Lipo-β-E are stable at 4 ***°***C for 30 days. The reason is that the DSPE-PEG 2000 chain on the surface of the liposomes can prevent the accumulation of liposomes, providing stability of the formulation.

### Pharmacokinetics of PEG-Lipo-β-E

Pharmacokinetic studies were performed to compare PEG-Lipo-β-E to an elemene injection by determining the drug concentration in rat plasma up to 90 minutes after i.v. administration. The plasma concentration *vs.* time curves for the PEG-Lipo-β-E and elemene injection are shown in **Figure 5B**. The mean plasma concentrations decreased in both groups. At the first 5 time points, plasma β-E concentrations were higher in the PEG-Lipo-β-E group than in the elemene injection group. The main pharmacokinetic parameters examined in this study are shown in **Table 7**. Parameters, such as the mean residence time (MRT) and steady-state apparent volume of distribution (V_z_), showed no significant differences between the 2 groups (*P* > 0.05). However, compared to the elemene injection group, the PEG-Lipo-β-E group demonstrated a 1.75-fold decrease in clearance (CL_z_) and a 1.62-fold increase in half-life (T_1/2z_). In addition, the area under the concentration-time curve (AUC) after injection of PEG-Lipo-β-E was 1.76 times higher than after elemene injection administration (*P* < 0.05). The higher AUC, longer half-life and slower clearance of PEG-Lipo-β-E compared to the elemene injection demonstrated that the availability of β-E is increased by forming PEGylated liposomes.

### *In vitro* cytotoxicity studies in human bladder cancer cells 

**Figure 6:**
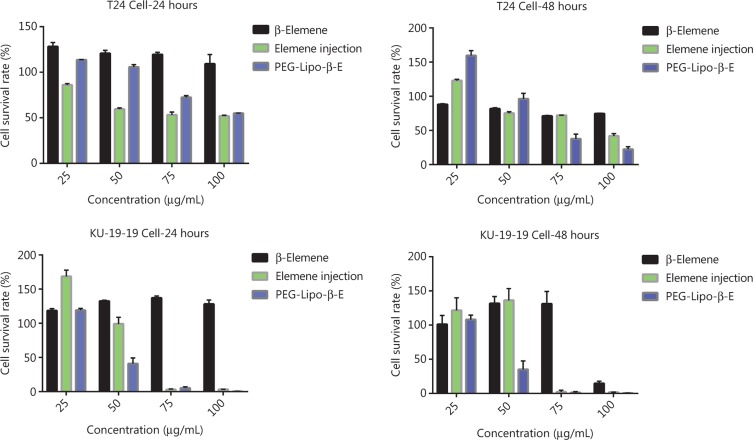
*In vitro* cytotoxicity of β-E, PEG-Lipo-β-E and elemene injection on T24 cells and KU-19-19 cells at 24 hours and 48 hours by CCK-8 assay.

A CCK-8 assay was used to evaluate the effect of free β-E, elemene injection and PEG-Lipo-β-E on cell proliferation in T24 cells and KU-19-19 cells (**Figure 6**). A concentration-dependent decline in the percent of cell survival was observed upon treatment with 2 preparations at all the studied time points. After 24 hours of incubation, we found that free β-E could promote cell growth at concentrations of 25, 50, 75 and 100 µg/mL in T24 cells and KU-19-19 cells. The inhibition rate of 100 µg/mL free β-E in T24 cells was 25.5%, while the IC_50_ of free β-E in KU-19-19 cells was 97.27 µg/mL after 48 hours of incubation. The IC_50_ value of the elemene injection in T24 cells was 91.41 and 92.90 µg/mL at 24 hours and 48 hours, respectively, while the IC_50_ of PEG-Lipo-β-E in T24 cells was 102.33 and 72.78 µg/mL at 24 hours and 48 hours, respectively. Similarly, in KU-19-19 cells, the IC_50_ value of the elemene injection was 63.24 and 71.45 µg/mL at 24 and 48 hours, respectively, whereas the IC_50_ value of PEG-Lipo-β-E was 48.19 and 47.53 µg/mL at 24 and 48 hours of incubation, respectively.

Compared to 24 hours of incubation, the IC_50_ value of the elemene injection showed a slight increase in T24 cells and a 1.13-fold increase in KU-19-19 cells at 48 hours, while the PEG-Lipo-β-E demonstrated a 1.41-fold decrease in IC_50_ in T24 cells and a slight decrease in KU-19-19 cells at 48 hours of incubation. PEG-Lipo-β-E showed more cytotoxicity at 48 hours, and the number of viable T24 cells and KU-19-19 cells was found to be less after incubation with PEG-Lipo-β-E. Compared to elemene injection, although the PEG-Lipo-β-E demonstrated a 1.12-fold increase in the IC_50_ in T24 cells at 24 hours, the IC_50_ value was 1.28-fold less at 48 hours; in KU-19-19 cells, the PEG-Lipo-β-E showed a 1.31- and 1.50-fold decrease in IC_50_ at 24 hours and 48 hours, respectively. β-E in the free and encapsulated forms exhibited better cytotoxicity against KU-19-19 cell lines compared to T24 cell lines. These data suggest a prolonged anticancer effect of PEGylated liposomes when compared with free β-E and conventional injection.

### *In vitro* cell migration assay

**Figure 7:**
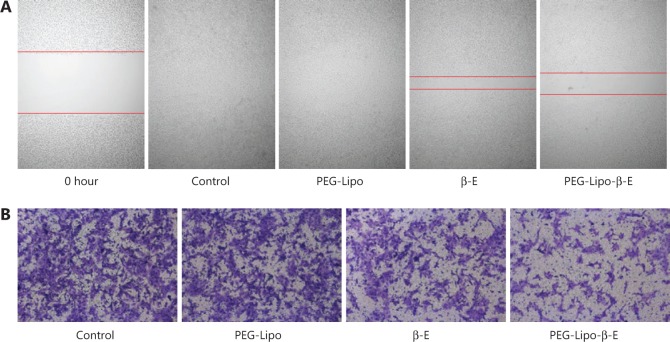
Typical images of wound healing and Transwell migration of PEG-Lipo, free β-E and PEG-Lipo-β-E (30 µg/mL) at 36 hours in KU-19-19 cells. (A) Wound healing (4 ×); (B) Transwell migration (10 ×), cells were stained by crystal violet staining solution.

As cell migration was closely related to tumor metastasis, cell motility was examined by wound healing and Transwell migration assays. An aggressive bladder cancer cell line, KU-19-19, was selected for this experiment (**Figure 7**). The CCK-8 assay showed that 30 µg/mL β-E did not exert obvious cytotoxicity under the tested cell conditions. In the control group, the forming scratch gap was barely observed after 36 hours, indicating that the KU-19-19 cells had strong motility. PEG-Lipo did not affect cell migration. Free β-E and PEG-Lipo-β-E could prevent cell motility, and the wound closure of the PEG-Lipo-β-E group could be greatly reduced after 36 hours. In the Transwell migration assay, there was almost no inhibitory effect from PEG-Lipo, and the inhibitory effect of free β-E was slight. However, a significant inhibitory effect was observed in the PEG-Lipo-β-E group. These results suggest that PEG-Lipo-β-E not only enhances cell cytotoxicity but also inhibits cell migration.

### *In vivo* antitumor efficacy

**Figure 8:**
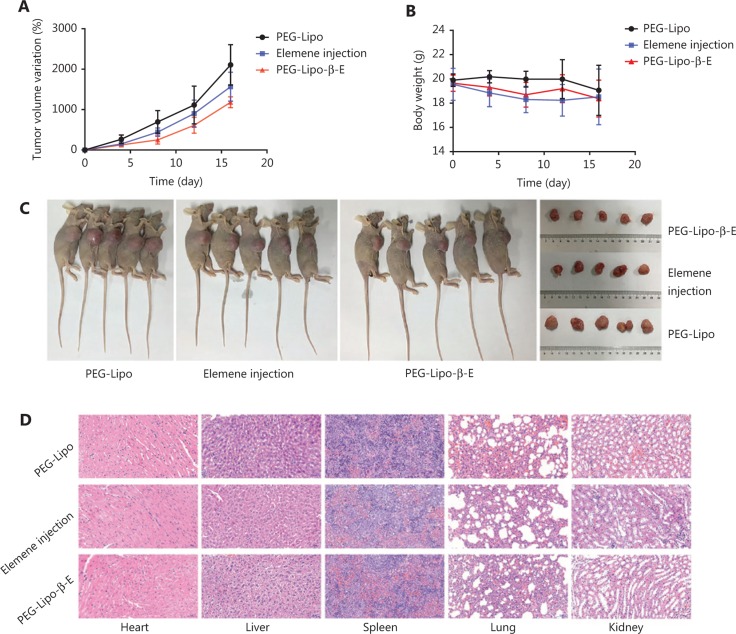
*In vivo* antitumor effect and safety evaluation of PEG-Lipo-β-E and elemene injection. (A) Variation of TV after treatment with PEG-Lipo, elemene injection and PEG-Lipo-β-E at a dose of 50 mg/kg after 16 days. (B) Body weight changes during treatment with different formulations. (C) Photographs of KU-19-19 bladder tumor-bearing BALB/c nude female mice and tumors. (D) Histological analysis (20 ×) of different organs after treatment with different formulations.

The antitumor effect of PEG-Lipo-β-E was evaluated in KU-19-19 bladder tumor-bearing BALB/c nude female mice by assessing the TV variation over time. As shown in **Figure 8A** and **8C** and **Table 8**, the TV in the blank liposome treatment group increased rapidly over time. In contrast, the RTV was significantly lower in mice treated with elemene injection and PEG-Lipo-β-E at doses of 50 mg/kg. In addition, TGI was higher with PEG-Lipo-β-E treatment (45.67%) compared to elemene injection (33.10%) (*P* < 0.05). These results suggest that the antitumor efficacy of PEG-Lipo-β-E is enhanced, an effect that may be attributed to increased bioavailability.

### Safety evaluation

The body weight variation was evaluated during treatment as an indicator of toxicity. As seen in **Figure 8B**, although a slight body weight loss was observed in the elemene injection and PEG-Lipo-β-E group compared with the blank liposome treatment group, there were no significant differences (*P* > 0.05), indicating the good safety profiles of the nanoparticles. The elemene injection group also showed a slight weight loss than the PEG-Lipo-β-E group, indicating that the PEG-Lipo-β-E might have better safety than the elemene injection. In addition, histological analysis of different organs was performed at the end of the treatment period (**Figure 8D**), no significant pathological features such as endothelial cell damage, necrosis, inflammatory response, and regenerative changes were observed in the experimental group compared with the control group. This evaluation revealed no evidence of toxicity to the heart, kidney, liver, lung, or spleen of the animals.

## Discussion

The results of the present study demonstrate that PEGylated β-E liposomes were successfully developed with a pH of 6.65 and demonstrated good stability after being stored for 30 days. As biodegradable and essentially nontoxic vesicles, stealth liposomes have been used as delivery systems for many antitumor drugs^31,48–50^. In the present study, PEGylated liposomes were prepared by ethanol injection and high-pressure micro-jet homogenization. This method is simple and rapid, and the possibility of denaturation of sensitive components is small. This method is mild to lipids and encapsulated materials, and the volume of ethanol in the medium is only 2%, which can reduce residual organic solvents. To improve the stability of liposomes, the oxidative degradation of the liposome component phospholipids should be prevented during preparation. The hydrolysis of soybean phospholipids is mainly catalyzed by hydrogen ions and hydroxide ions, and the hydrolysis rate is the smallest at a pH of approximately 6.5^51^. In this study, L-histidine was used instead of disodium hydrogen phosphate and sodium dihydrogen phosphate in the conventional elemene injection to adjust the pH of the solution, on the one hand, to reduce the hydrolysis of soybean phospholipids, and on the other hand, to reduce irritation of the liposomes.

FTIR and DSC are commonly available tools to provide information about the physicochemical state of liposomes. In order to prevent the influence of water, The PEG-Lipo-β-E was freeze-dried before measurement. During the thermal analysis, we also investigated the heat flow changes of β-E at 0 to 80 °C. β-E has no endothermic peaks and an exothermic peak because β-E is an oily liquid at normal temperature. Therefore, we verified the formation of liposomes by comparing liposome materials, as well as the formation of blank and drug-containing liposomes. The results showed that the phase transition temperature was increased after the formation of liposomes for β-E, and the heat flow changed significantly.

Both gas chromatography and liquid chromatography can be used for the determination of β-E. We found that the quantitative results obtained by gas chromatography were inaccurate and the repeatability was poor, probably because β-E was easily volatilized. UFLC could improve the separation effect, speed up the detection, and facilitate batch and rapid analysis. The detection time of liquid chromatography is 4 times shorter than that of gas chromatography. In the test, methanol:water (80:20) was used as the mobile phase, but the baseline was unstable and there was a tailing phenomenon. The mobile phase was adjusted to be acetonitrile-water, which could obviously improve the peak shape and separation. In the process of plasma samples treatment, by comparing the methanol and acetonitrile, we found that acetonitrile could precipitate proteins better. In the determination of the plasma sample content, we found that the concentration of the drug in the sample was not high after adding 1.3 mL of acetonitrile to 0.2 mL of plasma. Therefore, we adjusted the ratio of plasma to acetonitrile (1:4) to obtain a larger amount of β-E for detection. During the processing of plasma samples, it is often necessary to add internal standards to eliminate errors in sample pretreatment and determination. The UFLC method used in this study had been reported in a related study^37^. The recovery and matrix effects of the sample after simple protein precipitation by acetonitrile meet the requirements. Therefore, we did not quantify using the internal standard method, which is not rigorous in this study. This method needs to be improved in subsequent studies to ensure the accuracy of the measurement.

β-E is a water-insoluble volatile oil with a lighter density than water. The free β-E in the liposomes should float on the surface of the water. We had not been able to separate and determine free β-E by Sephadex column chromatography as the insoluble β-E floating on water might be adsorbed by the gel particles, resulting in recovery that did not meet the requirements. Therefore, the EE of PEG-Lipo-β-E was obtained by determining the drug concentration of the internal and total solutions using the liquid surface method^37^.

The higher AUC, longer half-life and slower clearance of PEG-Lipo-β-E compared to the elemene injection demonstrated that the availability of β-E is increased by forming PEGylated liposomes. The PEGylated liposome exhibited a slow release as the loaded drug required a transfer from the PEG to the aqueous phase. The mechanism might be that DSPE-PEG 2000 produced a nano-thickness steric layer on the surface of the liposome. This sterically hindered layer acts similar to a “brush” that excludes other macromolecules or lipoprotein complexes away from the liposome, thereby weakening the effects of various components of the blood, especially the effect of blood plasma opsonins, and subsequent RES uptake. Furthermore, DSPE-PEG 2000 has a long polar group that increases the hydrophilicity of the liposome surface, thereby reducing the rate and extent of liposome uptake by the mononuclear phagocytic system (MPS). At the same time, this long polar group effectively prevents the surface of the liposomes from interacting with plasma albumin and reduces the affinity of the liposomes to MPS^52,53^. Thus, PEG-Lipo-β-E produced higher plasma concentrations.

From the results of *in vitro* cytotoxicity studies in human bladder cancer cells, it is evident that the cytotoxic effects of free β-E at 24 or 48 hours of treatment are not good, especially at 24 hours of incubation, which may be due to the volatility and poor bioavailability of the pure drug. The promotion of cell growth might be because the surviving fraction of cells in the population are able to proliferate in the cell culture at longer incubation times and in turn increase the percent of viable cells. However, for the 2 preparations, the 24- and 48-hour assays showed a significant reduction in the cell survival rate when compared with the pure drug. Moreover, it is striking to note that the cytotoxic effects of PEG-Lipo-β-E significantly increased, and the percent of viable cells remained lower compared to the elemene injection. This remarkable enhancement in cytotoxicity is due to the slow release of the drug from DSPE-PEG 2000. The anticancer effects of PEG-Lipo-β-E remained constant or increased for the longer incubation time (48 hours), but in the case of the conventional injection, a decline in the activity was observed after 48 hours of incubation.

The evaluation of the antitumor effect of treatment with PEG-Lipo-β-E in a bladder cancer xenograft model showed that the TGI of PEG-Lipo-β-E is enhanced. Elemene injection and PEG-Lipo-β-E could target of tumors via the enhanced permeability and retention effect. After the common elemene injection entered the body, it was quickly removed from the body, making it difficult to exert a better anti-tumor effect. Because of the existence of the stealth component (DSPE-PEG 2000), long-circulating liposomes had prolonged retention time in the body, which meant β-E had more opportunities to passively target tumors, and the curative effect was improved. The safety of PEG-Lipo-β-E was also evaluated by body weight variation and histological analysis of different organs. Compared with chemotherapy drugs, β-E is a noncytotoxic antitumor drug extracted from the herb *Curcumae Rhizoma*. There was no evidence of toxicity to the heart, kidney, liver, lung, or spleen of the animals in PEG-Lipo-β-E group by histological analysis. Moreover, there was no obvious weight loss in the PEG-Lipo-β-E group than the PEG-Lip group, indicating that the safety was good. Therefore, the results from this study suggest the potential applicability of PEG-Lipo-β-E as a new and promising anticancer formulation.

## Conclusions

The present study demonstrates the PEG-Lipo-β-E as a new formulation with ease of preparation, high EE, good stability, improved bioavailability and anti-tumor effects. Therefore, the PEGylated β-elemene liposome may have potential uses as an anticancer drug formulation in the future.

## Figures and Tables

**Table 1:** Intra-day and inter-day precision and accuracy of β-E in rat plasma (*n* = 5)

Nominal concentration (μg/mL)	Precision (%)		Accuracy (%)	
	Intra-day	Inter-day	Intra-day	Inter-day
LQC (0.6)	2.0	2.2	103.8	105.0
MQC (2)	1.2	1.8	97.3	100.4
HQC (10)	2.3	2.1	99.5	99.5

LQC, low quality control; MQC, middle quality control; HQC, high quality control.

**Table 2:** Mean extraction recoveries and matrix effects of β-E in rat plasma (*n* = 5)

Nominal concentration (μg/mL)	Calculated concentration (μg/mL)	Recovery (%)	Matrix effect (%)
LQC (0.6)	0.6 ± 0.1	97.0 ± 2.1	96.3 ± 6.0
MQC (2)	1.9 ± 0.1	96.6 ± 1.2	99.33 ± 1.1
HQC (10)	10.4 ± 0.2	101.6 ± 2.3	101.6 ± 1.3

LQC, low quality control; MQC, middle quality control; HQC, high quality control.

**Table 3:** Stability (%) of β-E in rat plasma (*n* = 5)

Nominal concentration (μg/mL)	Room temperature^a^	Autosampler^b^	Freeze-thaw^c^	*−*20 °C for 48 hours
LQC (0.6)	93.7 ± 1.0	94.8 ± 2.8	96.38 ± 1.0	98.0 ± 3.0
MQC (2)	97.1 ± 0.9	99.6 ± 2.3	95.9 ± 0.7	101.0 ± 3.4
HQC (10)	95.4 ± 0.5	103.1 ± 2.2	98.99 ± 4.6	103.37 ± 0.3

LQC, low quality control; MQC, middle quality control; HQC, high quality control; ^a^room temperature during 4 hours; ^b^25 °C during 24 hours in the autosampler; ^c^3 freezing and thawing cycles.

**Table 4:** Durability (%) of β-E in rat plasma (*n* = 3)

Analytes	Method parameter	Durability (%)
MQC (2)	Flow rate: 0.98 mL/minute	98.5 ± 2.2
	Flow rate: 1.02 mL/minute	93.0 ± 0.5
	Acetonitrile/water (78:22)	94.0 ± 3.4
	Acetonitrile/water (82:18)	101.4 ± 1.9
	Temperature 38 °C	95.6 ± 1.0
	Temperature 42 °C	94.7 ± 0.3

MQC, middle quality control.

**Table 5:** Nonlinear fits of β-E release from elemene injection and PEGylated liposomes

Equation type	Elemene injection		PEG-Lipo-β-E	
	Equation	*R*^2^	Equation	*R*^2^
Zero-order model, *F* = *K*_0_ * *t*	*F* = 3.150 * *t*	0.3520	*F* = 2.289 * *t*	0.6869
First-order model, *F* = 100 * (1 – *e*^−*k*_1_*t^)	*F* = 100 * (1 – *e*^−0.101*t^)	0.9375	*F* = 100 * (1 – *e*^−0.042*t^)	0.9301
Higuchi model, *F* = *K*_*H*_ * *t*^0.5^	*F* = 16.839 * *t*^0.5^	0.8838	*F* = 11.913 * *t*^0.5^	0.9836
Weibull model, 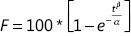	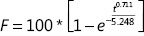	0.9511	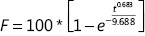	0.9909

*F*, the fraction (%) of drug released in time *t*; *K*_0_, *K*_1_ and *K_H_*, the zero-order, first-order, and Higuchi release constant, respectively; α, the scale parameter which defines the time scale of the process; β, the shape parameter which characterizes the curve as either exponential (β = 1; case 1), sigmoid, S-shaped, with upward curvature followed by a turning point (β > 1; case 2), or parabolic, with a higher initial slope and after that consistent with the exponential (β < 1; case 3).

**Table 6:** Entrapment efficiency, mean particle size, appearance and β-E content of PEG-Lipo-β-E formulations at 4 °C (*n* = 3)

Time (days)	Appearance	Entrapment efficiency (%)	Particle size (nm)	β-E content (mg/mL)
0	Uniform	95.53 ± 1.712	83.31 ± 0.181	5.024 ± 0.107
7	Uniform	92.61 ± 0.782	83.25 ± 0.217	5.018 ± 0.091
15	Uniform	92.93 ± 1.542	83.83 ± 1.418	4.985 ± 0.043
30	Uniform	91.44 ± 3.926	85.09 ± 1.701	4.983 ± 0.018

**Table 7:** Pharmacokinetic parameters in rats after intravenous administration of PEG-Lipo-β-E and elemene injection

Parameters	Units	Elemene injection	PEG-Lipo-β-E
AUC_(0-t)_	mg/L*min	69.951 ± 14.118*	123.331 ± 3.265*
MRT_(0-t)_	min	17.175 ± 1.591	15.989 ± 0.599
T_1/2z_	min	15.964 ± 1.938*	25.850 ± 0.609*
V_z_	L/kg	0.428 ± 0.219	0.258 ± 0.046
CL_z_	L/min/kg	0.014 ± 0.003*	0.008 ± 0.000*

The data are represented as the mean ± SD (*n* = 5). AUC, area under the concentration-time curve; MRT, mean residence time; T_1/2z_, half-life; V_z_, steady-state apparent volume of distribution; CL_z_, clearance. *P < 0.05 was considered statistically significant.

**Table 8:** RTV and TGI after administration of PEG-Lipo, elemene injection and PEG-Lipo-β-E

Treatment	RTV	TGI (%)
PEG-Lipo	22.06 ± 5.02	–
Elemene injection 50 mg/kg	16.65 ± 3.09*	33.10
PEG-Lipo-β-E 50 mg/kg	12.82 ± 1.36*	45.67

The data are represented as the mean ± SD (*n* = 5). RTV, relative tumor volume; TGI, tumor growth inhibition. *P < 0.05 was considered statistically significant.
