# Supplementary material for: Preparation, characterization, pharmacokinetics and anticancer effects of PEGylated β-elemene liposomes
Source: Cancer Biol Med. 2020 Feb 15;17(1):60–75. doi: 10.20892/j.issn.2095-3941.2019.0156 (PMC7142831; doi:10.20892/j.issn.2095-3941.2019.0156)
Supplement: Supplementary file 1 [file cbm-17-060-s001.pdf]

Supplementary materials

Table S1 Calibration curves, linear ranges, correlation coefficients, and LLOQ of β-E in PEGylated liposomes and rat plasma (n = 5)

Analytes	Linear regression equation	R^2	Linear range (μg/mL)	LLOQ (μg/mL)	Accuracy (%)	Precision (%)
PEG-Lipo-β-E	y = 3806.97886 x + 113.22407	0.9998	0.6~50.4	0.6	104.5	3.2
β-E in rat plasma	y = 7486.41884 x + 385.39760	0.9999	0.6~20.5	0.6	94.1	4.75

β-E, β-elemene; LLOQ, lower limit of quantification

Table S2 Intra-day and Inter-day precision and accuracy of β-E in PEGylated liposomes (N = 5)

Analytes	Precision (%)		Accuracy (%)	
	Intra-day	Inter-day	Intra-day	Inter-day
QC (20)	0.25	0.75	99.8	100.4
Sample (20)	0.93	2.33	98.38	100.6

QC, quality control

Table S3 Mean recoveries of β-E in PEGylated liposomes (n = 5)

Analytes	Nominal concentration (μg/mL)	Calculated concentration (μg/mL)	Recovery (%)
Sample (20)	10.1	10.0 ± 0.1	95.0 ± 0.1
	20.1	20.0 ± 0.1	97.1 ± 0.1
	30.2	29.7 ± 0.2	97.5 ± 0.1

Table S4 Stability (%) of β-E in PEGylated liposomes (n = 5)

Analytes	Room temperature for 24 hours	Room temperature for 48 hours
QC (20)	102.4 ± 0.1	102.8 ± 0.3
Sample (20)	99.6 ± 0.5	100.2 ± 0.4

QC, quality control

Table S5 Durability (%) of β-E in PEGylated liposomes (n = 3)

Analytes	Method parameter	Durability (%)
QC (20)	Flow rate: 0.98 mL/minute	100.8 ± 0.1
	Flow rate: 1.02 mL/minute	97.6 ± 0.3
	Acetonitrile/water (78:22)	100.8 ± 0.1
	Acetonitrile/water (82:18)	100.6 ± 0.2
	Temperature 38 °C	100.1 ± 0.2
	Temperature 42 °C	100.1 ± 0.2
Sample (20)	Flow rate: 0.98 mL/minute	100.2 ± 0.1
	Flow rate: 1.02 mL/minute	96.3 ± 0.2
	Acetonitrile/water (78:22)	98.0 ± 0.1
	Acetonitrile/water (82:18)	96.9 ± 0.2
	Temperature 38 °C	98.1 ± 0.2
	Temperature 42 °C	97.8 ± 0.1

QC, quality control
